# Cross-Cultural Adaptation and Validation of SNOT-20 in Portuguese

**DOI:** 10.1155/2011/306529

**Published:** 2011-05-03

**Authors:** Thiago Freire Pinto Bezerra, Jay F. Piccirillo, Marco Aurélio Fornazieri, Renata R. de M. Pilan, Tatiana Regina Teles Abdo, Fabio de Rezende Pinna, Francini Grecco de Melo Padua, Richard Louis Voegels

**Affiliations:** ^1^Department of Otorhinolaryngology and Ophthalmology, School of Medicine, University of São Paulo, São Paulo, SP, Brazil; ^2^Division of Clinical Outcomes Research, Department of Otolaryngology-Head and Neck Surgery, Washington University School of Medicine, St. Louis, MO 63110, USA

## Abstract

*Introduction*. Chronic rhinosinusitis is a highly prevalent disease, so it is necessary to create valid instruments to assess the quality of life of these patients. The SNOT-20 questionnaire was developed for this purpose as a specific test to evaluate the quality of life related to chronic rhinosinusitis. It was validated in the English language, and it has been used in most studies on this subject. Currently, there is no validated instrument for assessing this disease in Portuguese. *Objective*. Cross-cultural adaptation and validation of SNOT-20 in Portuguese. *Patients and Methods*. The SNOT-20 questionnaire underwent a meticulous process of cross-cultural adaptation and was evaluated by assessing its sensitivity, reliability, and validity. *Results*. The process resulted in an intelligible version of the questionnaire, the SNOT-20p. Internal consistency (Cronbach's alpha = 0.91, *P* < .001), reliability testing-retesting (*r* = 0.994, *P* < .001), content validity, validity of discrimination of patients without chronic rhinosinusitis (*U* = 44, *P* < .0001) and assessment of sensitivity to change (SRM = 1.53 and 1.09) were evaluated. *Conclusion*. We conducted a successful process of cross-cultural adaptation and validation of the SNOT-20 questionnaire into Portuguese.

## 1. Introduction

Rhinosinusitis (RS) is one of the most common complaints presented in physician office visits. About 31 million people are affected in the United States (USA) each year, with an annual cost of six billion dollars. It is one of the main reasons for prescribing antibiotics and lost worker productivity [1–3]. RS that lasts more than 12 weeks is called chronic rhionosinusitis. The Chronic rhinosinusitis is divided into with and without nasal polyps, distinguished by clinical examination, histopathology, and prognosis profile of interleukin [4]. The chronic RS is also a common reason for surgery, with more than 200,000 sinusectomies held each year in the USA [5].

Specific instruments to measure quality of life related to RS have been developed by the need to better assess the morbidity of a disease and the evolution and impact of treatments. Measures of quality of life related to RS with validity and reliability are crucial for assessing treatment outcomes of CRS [6]. In the past, some studies demonstrating the benefits of antibiotics presented as the primary endpoint only the descriptive report of improvement of symptoms of the patient, for example. Quality of life is seen in a different state of health. It is the only personal experience that reflects not only the health, but also other factors and circumstances in the patient's life that only he can express [7].

The incorporation of a validated and standardized instrument for assessing quality of life in a study is very important because it allows comparison of results with other studies, depending on the instrument with other diseases [7]. The study entitled “Medical Outcomes Study Short-Form 36-Item Health Survey (SF-36)” shows any increased morbidity, in measures of bodily pain and social function in patients with RS more than for patients with congestive heart failure, angina, chronic obstructive pulmonary disease, or back pain, suggesting a far greater impact than was currently assessed for the RS [8].

A health-related and disease-specific instrument for quality of life evaluation called “20-Item Sino-Nasal Outcome Test” was created and validated for use in the English language, and its use is widespread in the literature by the countries of this language [6]. Known as SNOT-20 (Annex I), it has been validated for use in German [9]. The adaptation and validation in our language is not only essential for its proper use but also allows the comparison of our results with published results in all other languages in which the same questionnaire was validated.

No version of this questionnaire of quality of life has yet been validated for use in Portuguese. A Portuguese version would give us better opportunities for evaluation of patients in studies and more accurate comparison of results with other treatments.

## 2. Objective

Validation and cross-cultural adaptation of the SNOT-20 (Sino-Nasal Outcome Test) in Portuguese is the objective of this paper.

## 3. Methods

### 3.1. Patients

This is a prospective cohort study conducted at Clinics Hospital, University of São Paulo after Ethics Committee approval. All patients agreed and signed informed consent.

**(I) Inclusion criteria**

- Patients with CRS, defined as the presence, for a period exceeding twelve weeks, of two or more of the following four symptoms, and one of these should be the first two: nasal blockage, obstruction or congestion, nasal discharge (anterior or posterior nasal drip), facial pain or pressure, and reduction or loss of smell [3];
- age of at least 18 years;
- good general health with no systemic or localized diseases that compromise or may compromise it.

**(II) Exclusion Criteria**

- Patients with secondary causes of RSC: fungal ball, invasive fungal disease, granulomatous diseases, vasculitis, isolated mucoceles, benign and malignant sinonasal tumors, congenital abnormalities (such as primary ciliary dyskinesia and cystic fibrosis), and oro-antral fistula;
- pregnancy and lactation;
- congenital craniofacial abnormalities;
- primary or secondary immunodeficiencies.

### 3.2. Translation and Cross-Cultural Adaptation

 We conducted the process of translation and cross-cultural adaptation from the original version of SNOT-20 instrument in English, following standardized rules for this purpose [10].

1. Two bilingual translators whose native language was Portuguese produced two separate versions of the instrument in this language: version T1 (translator with medical knowledge) and version T2 (translator without medical knowledge).
2. The two versions were synthesized into a final version in Portuguese (SNOT-20p version) through a meeting with the two translators and one outside observer with ENT background.
3. The SNOT-20p was backtranslated into English (BT version) by two bilingual translators with English as their native language (BT1 and BT2 versions) independently. The new version in English version (BT) was synthesized from a meeting with the two translators and one outside observer with ENT background.
4. The six versions were sent to the author of the original English version, so that he could certify that the original meaning of the questionnaire had been maintained and to authorize the start of the validation process in Portuguese.

### 3.3. Validation

Patients with chronic rhinosinusitis who underwent endoscopic sinus surgery responded to the questionnaire in four stages as follows: fifteen days before the surgery, the day before surgery, six months after surgery, and twelve months after surgery.

### 3.4. Statistical Analysis

Internal consistency reliability was estimated by Cronbach's alpha [11] and test-retest reliability of the questionnaire about fifteen days after the first evaluation (Wilcoxon signed-rank test and Spearman's rank correlation coefficient). Discriminant validity was estimated by comparing total scores of the results between two different groups of the department of otolaryngology, patients with CRS, and patients with appointments for other reasons and without clinical conditions compatible with CRS (Mann-Whitney *U*-test). Sensitivity for clinical change was calculated by standardized response mean (SRM). We also evaluated the mean SNOT-20 score and mean SNOT-20 score for five important items and variations collected after six and twelve months (Friedman test and Wilcoxon signed-rank test).

An alpha of 0.05 was considered statistically significant for all statistical tests. Data analysis was performed using SPSS 10.0 (SPSS Inc, Chicago, IL).

## 4. Results

This study was conducted in a tertiary care center from February 2008 to October 2010. The SNOT-20 questionnaire was answered by 38 patients with median ages of 42.5 ± 18.2 (20–76) years (median (md) ± interquartile range (IQR)), of which 63% were men. All patients completed the questionnaire before surgery, but only 32 responded after 6 months, and only 34 after 12 months of surgery. The SNOT-20 scores preoperatively and after 6 and 12 months of surgery were 1.75 (±2.04), 0.68 (±1.07), and 0.90 (±1.64) (md (±IQR)), Friedman Test, *P* < .0001. The items most commonly chosen as the worst in the preoperative and the percentage selected were “Need to blow nose” (57.9%), “Sneezing” and “Postnasal discharge” (42.1%), and “Thick nasal discharge,” “Difficulty falling asleep,” and “Wake up tired” (31.6%). 

The Cronbach's alpha was 0.91 (*P* < .001). Reliability was evaluated by test-retest and demonstrated by the high Spearman correlation coefficient (*r* = 0.994, *P* < .001). The average difference in SNOT-20 scores in evaluating the reliability of the testing-retesting was not statistically significant (Mean ranks (reproduction versus day before surgery) 6.92. versus 4.90, *P* = .448, Wilcoxon signed-rank test). 

The content validity of the questionnaire was ensured by how the original version was developed, and by literature review, by interviews with patients and discussion with experienced otolaryngologists. This thorough process aimed to help the maintenance of the practical purpose of this instrument. The instrument was sent to the author in English to prove that the original meaning had been maintained.

Twenty-five patients of the Otorhinolaryngology Department without sinonasal complaints responded to the questionnaire SNOT-20p. The median score of patients without sinonasal complaints was 0.39 ± 0.49 and suggested few complaints on the questionnaire. The validity of discrimination between patients with and without chronic sinusitis was confirmed by Mann-Whitney *t*-test that showed a statistically significant difference between groups (*U* = 44, *P* < .001).

The SRM was 1.53 and 1.09, six and twelve months after surgery, respectively. These results suggested a high sensitivity to change for both measures.

The Wilcoxon signed-rank test showed that the difference between the median SNOT-20p on 5 items selected as most important for each patient and the median overall score was higher and statistically significant in the initial visit (3.97 ± 1.03 (md ± IQR) versus 2.16 ± 1.17, (*P* < .001), after 6 months (1 . 33 ± 1.12 (md ± IQR) versus 0.89 ± 0.80, (*P* < .001)), and after 1 year (1.53 ± 1.36 (md ± IQR) versus 1.16 ± 1.01, (*P* < .002)). The change in SNOT-20 questionnaire for the important items was also statistically higher than the average overall score after 6 months (−2.76 ± 1.07 (md ± IQR) versus −1.40 ± 0.84, (*P* < .001)) and after 1 year (−2.46 ± 1.37 (md ± IQR) versus −1.05 ± 0.87, *P* < .001)). The SRM for these most important items was higher after six months (2.42) and 1 year after surgery (1.74).

## 5. Discussion

This paper concludes the process of cross-cultural adaptation and validation of disease-specific QOL questionnaire SNOT-20 for Portuguese-speaking physicians to evaluate patients with CRS. The process of translation and cross-cultural adaptation was completed without any difficulty and resulted in a questionnaire version in Portuguese, the SNOT-20p, with the contents maintained. The process ensures that any study that uses the Portuguese questionnaire SNOT-20p and makes reference to this paper of validation can publish and compare the results with the SNOT-20 questionnaire used in any other study in English or another language that has validated the questionnaire.

Internal consistency (Cronbach's alpha = 0.91, *P* < .001), reliability of test-retest (*r* = 0.994, *p* < .001), content validity, validity of discrimination with patients without chronic rhinosinusitis (*U* = 44, *P* < .001), and assessment of sensitivity to change (SRM = 1.53 and 1.09) were tested during the validation process. It was also shown how the five items chosen by the patient as most important are more sensitive in detecting changes in this instrument than the value of the overall score (SRM = 2.42 and 1.74).

The instruments that assess the disease-specific quality of life for some disorders are necessary because sometimes the changes caused by them can be small or specific to certain disease that instruments to assess the global health or specific symptoms could not identify [12].

There are many other instruments available and validated to assess the impact of rhinosinusitis on quality of life, its evaluation and impact in the treatment. The RSOM-31 (from the English “Rhinosinusitis outcome measure") contains 31 questions divided into seven areas; however the scale at which the patients were required to answer, was a little difficult to interpret [13]. The RSDI (from the English “Rhinosinusitis Disability Index”) relates sinonasal symptoms to specific limitations in daily life also through 30 questions, similar to RSOM [14]. The RQLQ (from the English “Rhinoconjunctivitis Quality of Life Questionnaire”) is a questionnaire for allergic symptoms and is not validated for sinusitis [15].

The SNOT-20 is a modified version of RSOM-31 in which eleven items were removed either by their redundancy, or because they do not contribute significantly to the instrument. Furthermore, the fashion of the score of responses and the composition of the result of the questionnaire has been simplified, and the five items considered most important compose another score to analyze the results in complaints more uncomfortable for the patient [7].

All these instruments were validated and applied in prospective studies in rhinosinusitis. The SNOT-20 is chosen as an outcome measure in most studies published on rhinosinusitis. We chose to validate the SNOT-20 because it is an instrument that assesses several important dimensions and specific quality of life of patients with chronic rhinosinusitis. Most patients in our pilot study responded quickly and easily to it. This instrument will allow us to generalize the results of national studies and compare them with other international studies.

The SNOT-22 is a recently validated instrument [16] formed by the simple addition of the evaluation of smell and nasal obstruction in SNOT-20. It is an important tool that has its role, but we believe that in most prospective studies it would be more precise to evaluate smell by specific tests, like the UPSIT [3]. Likewise, it is more specific to assess different aspects of nasal obstruction with the NOSE questionnaire [12]. Let us consider the other sides of the nasal obstruction because the patient often does not bother with these complaints but complains of nasal congestion or difficulty of breathing through his or her nose.

We chose to use these simple and rapid application tools in our studies because they evaluate these complaints in a meticulous fashion. We used a validated version of the NOSE questionnaire in Portuguese which has already been accepted for publication (the English “Nasal Obstruction Symptom Evaluation”) (Bezerra et al.) to assess the specific quality of life related to nasal obstruction. It could be used to evaluate any nasal disease related to that complaint, not only chronic rhinosinusitis. The UPSIT allows a broader assessment of hyposmia than a single question to the patient. The completion of the validation of the UPSIT test (University of Pennsylvania Smell Identification Test) for the Portuguese by Fornazieri et al. will allow an objective and specific assessment of the sense of smell in our Brazilian patients. The instrument already in use in Brazil contained some cultural differences, and the adaptation process will seek to produce a version closer to our culture.

The reliability of an instrument could be assessed by its internal consistency and the maintenance of the score over time when the patient's condition does not change. The internal consistency of the SNOT-20 assesses how each item relates to the instrument against the other items and against the final score. The Cronbach's alpha of 0.91 demonstrates a high internal consistency and a level necessary for clinical application (>0.9). The test-retest reliability is achieved when we apply the instruments at different times and analyze the correlation between the scores, which was also demonstrated by the questionnaire (*r* = 0.994, *P* < .001) [7].

The content validity of the questionnaire was ensured by the methodology used in developing the questionnaire as described above. There were four independent translations and two synthetic ones in a process of translation and retranslation. We emphasize that methodology allowed an adaptation process of the local version of the questionnaire according to international standards and maintaining the original meaning [10]. The participation of the author of the original questionnaire at this stage ensured the maintenance of the meaning of the instrument. The process could have generated a simple translation tool that was not equivalent to the original questionnaire and in capable of comparing responses across populations and generalization of data.

The importance of the patient choosing five items considered most important could be shown by more significant results detected in these items than on the overall score. The assessment of these five items, about which patients care most, demonstrated how these items have changed. The data also showed that the evaluation of outcome in these five items is much more sensitive to change than the overall questionnaire.

The validation of the SNOT-20 questionnaire for the Portuguese language opens up new frontiers for the generalization of data from our research, so now we can compare results with research from other countries in a much more accurate way. It is demonstrated to be a valid instrument for assessing what it intended to assess and to be highly sensitive to changes because the score of the instrument changes when the patient's condition also changes. It also showed a significant discrimination between patients with and without chronic rhinosinusitis. It is a valuable tool, and together with the NOSE questionnaire and UPSIT test, it allows the complete evaluation of nasal or sinonasal complaints in our patients. It could be used to compare disease-specific quality of life before and after treatments, between different groups of patients, to compare different treatments and to compare surgical techniques.

## Supplementary Material

Cross-cultural adapted and validated version of SNOT-20 in portuguese

## References

[1] Benninger MS, Ferguson BJ, Hadley JA (2003). Adult chronic rhinosinusitis: definitions, diagnosis, epidemiology, and pathophysiology. *Otolaryngology—Head and Neck Surgery*.

[2] Osguthorpe JD (2001). Adult rhinosinusitis: diagnosis and management. *American Family Physician*.

[3] European Academy of Allergology and Clinical Immunology (2005). European position paper on rhinosinusitis and nasal polyps. *Rhinol Supplement*.

[4] Voegels RL, De Melo Padua FG (2005). Expression of interleukins in patients with nasal polyposis. *Otolaryngology—Head and Neck Surgery*.

[5] Kennedy DW, Shaman  P, Han W, Selman H, Deems DA, Lanza DC (1994). Complications of ethmoidectomy: a survey of fellows of the american academy of otolaryngology—head and neck surgery. *Otolaryngology—Head and Neck Surgery*.

[6] Linder JA, Atlas SJ (2004). Health-related quality of life in patients with sinusitis. *Current Allergy and Asthma Reports*.

[7] Piccirillo JF, Merritt MG, Richards ML (2002). Psychometric and clinimetric validity of the 20-Item Sino-Nasal Outcome Test (SNOT-20). *Otolaryngology—Head and Neck Surgery*.

[8] Gliklich RE, Metson R (1995). The health impact of chronic sinusitis in patients seeking otolaryngologic care. *Otolaryngology—Head and Neck Surgery*.

[9] Baumann I, Blumenstock G, DeMaddalena H, Piccirillo JF, Plinkert PK (2007). Quality of life in patients with chronic rhinosinusitis. Validation of the Sino-Nasal Outcome Test-20 German Adapted Version. *HNO Journal*.

[10] Beaton DE, Bombardier C, Guillemin F, Ferraz MB (2000). Guidelines for the process of cross-cultural adaptation of self-report measures. *Spine*.

[11] Cronbach LJ (1951). Coefficient alpha and the internal structure of tests. *Psychometrika*.

[12] Stewart MG, Witsell DL, Smith TL, Weaver EM, Yueh B, Hannley MT (2004). Development and validation of the Nasal Obstructionsymptom Evaluation (NOSE) scale. *Otolaryngology—Head and Neck Surgery*.

[13] Piccirillo JF, Edwards D,  Haiduk A (1995). Psychometric and clinimetric validity of the 31-Item Rhinosinusitis Outcome Measure (RSOM-31). *American Journal of Rhinology*.

[14] Benninger MS, Senior BA (1997). The development of the rhinosinusitis disability index. *Archives of Otolaryngology—Head and Neck Surgery*.

[15] Juniper EF, Guyatt GH (1991). Development and testing of a new measure of health status for clinical trials in rhinoconjunctivitis. *Clinical and Experimental Allergy*.

[16] Hopkins C, Gillett S, Slack R, Lund VJ, Browne JP (2009). Psychometric validity of the 22-item sinonasal outcome test. *Clinical Otolaryngology*.

