# Supplementary material for: Cross-Cultural Adaptation and Validation of SNOT-20 in Portuguese
Source: Int J Otolaryngol. 2011 May 3;2011:306529. doi: 10.1155/2011/306529 (PMC3142655; doi:10.1155/2011/306529)
Supplement: Supplementary file 1 — Cross-cultural adapted and validated version of SNOT-20 in portuguese [file 306529.f1.pdf]

GRUPO DE RINOLOGIA DO HOSPITAL DAS CLÍNICAS DA USP

NOME: ______________________________________ SEXO: __

RGHC: ___________ IDADE: __ TELEFONE: (__) _____ - _____

AVALIAÇÃO DE RESULTADOS NASOSSINUSAIS. (SNOT-20)

Você encontrará abaixo uma lista de sintomas e conseqüências sociais e emocionais da sua rinossinusite. Gostaríamos de saber mais sobre estes problemas e pedimos que respondesse às seguintes perguntas da melhor maneira possível. Não há respostas certas ou erradas e somente você pode nos dar essas informações. Por gentileza, dê uma nota para seus problemas conforme se apresentaram nas duas últimas semanas. Obrigado por sua participação. Caso tenha alguma dúvida, peça a nossa ajuda imediatamente.

1. Considerando a gravidade do problema, quando aparece e a freqüência com que acontece; dê uma nota para cada item abaixo de acordo com o grau de intensidade do problema. Faça um círculo ao redor do número que corresponde ao que você sente. Use a escala ao lado:	Nenhum problema	Problema muito pequeno	Problema pequeno	Problema moderado	Problema sério	Pior problema possível	5 itens mais importantes
1. Necessidade de assoar o nariz	0	1	2	3	4	5	☐
2. Espirros	0	1	2	3	4	5	☐
3. Nariz escorrendo	0	1	2	3	4	5	☐
4. Tosse	0	1	2	3	4	5	☐
5. Secreção nasal escorrendo para a garganta	0	1	2	3	4	5	☐
6. Secreçao nasal grossa	0	1	2	3	4	5	☐
7. Ouvido tampado	0	1	2	3	4	5	☐
8. Tontura	0	1	2	3	4	5	☐
9. Dor de Ouvido	0	1	2	3	4	5	☐
10. Dor/ pressão na face	0	1	2	3	4	5	☐
11. Dificuldade de pegar no sono	0	1	2	3	4	5	☐
12. Acordar durante a noite	0	1	2	3	4	5	☐
13. Falta de uma boa noite de sono	0	1	2	3	4	5	☐
14. Acordar cansado	0	1	2	3	4	5	☐
15. Fadiga	0	1	2	3	4	5	☐
16. Produtividade diminuída	0	1	2	3	4	5	☐
17. Concentração reduzida	0	1	2	3	4	5	☐
18. Frustrado / Inquieto/ Irritado	0	1	2	3	4	5	☐
19. Triste	0	1	2	3	4	5	☐
20. Envergonhado com a doença	0	1	2	3	4	5	☐

2. Favor marcar os itens mais importantes que estão prejudicando sua saúde (máximo de 5 itens)

Bezerra, TFP; Piccirillo, JF; Fornazieri, MA; Pilan, RR de M; Abdo, TRT; Pinna, F de R; Padua, FG de M; Voegels, RV. (2011)
